# A community-based pharmacist-led smoking cessation program, before elective total joint replacement surgery, markedly enhances smoking cessation rates

**DOI:** 10.18332/tid/126405

**Published:** 2020-09-14

**Authors:** Lauren A. Beaupre, Fadi Hammal, Robert Stiegelmar, Edward Masson, Barry Finegan

**Affiliations:** 1 Department of Physical Therapy, University of Alberta, Edmonton, Canada; 2 Department of Surgery, University of Alberta, Edmonton, Canada; 3 Department of Anesthesiology and Pain Medicine, University of Alberta, Edmonton, Canada

**Keywords:** smoking cessation, surgery, community-based resources, pharmacy

## Abstract

**INTRODUCTION:**

We compared smoking cessation outcomes between those who used a pharmacist-led community-based smoking cessation intervention and those who did not, prior to total joint replacement (TJR) surgery. Also, we examined intervention characteristics (e.g. number/duration of sessions attended, recommended therapy) and smoking cessation outcomes.

**METHODS:**

This prospective evaluation was nested within a comparative study from a centralized clinic that prepares over 3000 patients annually for TJR and focused on participants referred to the community-based smoking cessation program preoperatively. Pharmacists offered an individualized evidence-based intervention and collected visit, duration and intervention data. Smoking cessation, the primary outcome, was ascertained independently of participating pharmacists at 6 weeks post-operative using exhaled CO monitoring and at 6 months post-recruitment via telephone interview.

**RESULTS:**

Of 286 eligible candidates, 104 agreed to participate, with one subsequently withdrawing (n=103). At 6 weeks post-operatively, 66/103 (64%) participants returned for study re-assessment while 63/103 (61%) participants completed the post-recruitment interview at 6 months; non-respondents to study follow-up were considered smokers. Of 103 participants, 58 (56%) consulted with a pharmacist; those who did not consult a pharmacist (n=45) were slightly younger (p=0.02) with significantly higher CO level (p=0.02) on study entry. Validated 7-day point prevalence abstinence (PPA) at 6 weeks post-operative was 11/58 (19%) in pharmacist-compliant participants compared to 2/45 (4%) in non-compliant participants (p=0.04). At 6 months post-recruitment, 19/58 (33%) pharmacistcompliant participants self-reported a 7-day PPA compared to 2/45 (4%) by non-compliant participants (p<0.001). For pharmacist-compliant participants, 33/58 (54%) saw the pharmacist 4 times; the mean overall pharmacist time was 71.8±24.4 minutes/patient with 26/58 (45%) and 19/58 (33%) prescribed nicotine replacement therapy and varenicline, respectively, and 13/58 (22%) not using medication; post hoc analysis suggested varenicline was marginally more effective for smoking cessation than no medication (p=0.04).

**CONCLUSIONS:**

Community-based pharmacist-led smoking cessation programs are an effective addition to usual preoperative care for smokers awaiting elective TJR. Using existing community resources led to higher smoking cessation rates in smokers waiting for TJR relative to those not using these resources.

## INTRODUCTION

Smoking cessation programs are not commonly used prior to elective surgeries despite evidence of their positive impact^1,2^. However, given the current smoking prevalence rate of 12% in Canada^3^, targeted smoking cessation approaches for those who are waiting for elective surgery are still needed. Over 60% of smokers scheduled for surgery who were offered a brief smoking cessation intervention in a surgical pre-admission clinic indicated that they would accept some form of cessation therapy^4^. Community pharmacists have recently been highlighted as effective providers of smoking cessation programs^5,6^. These programs are often readily available in the community but may not be considered part of standardized preoperative care for elective surgery. Rather than creating new programs within preoperative clinics, standardized referral processes to current community-based programs may facilitate preoperative smoking reduction and/ or cessation. This would achieve the aim of actively supporting surgical patients in achieving smoking reduction/cessation without substantially adding to the preoperative clinic workload.

We previously reported the effectiveness of inserting a fax referral process to an existing community pharmacist-led smoking cessation program before elective total joint replacement (TJR), relative to usual preoperative practice for smoking cessation^7^. This simple addition to the usual preoperative care process required very few clinic resources but resulted in a substantial increase in smoking cessation within 6 months of study recruitment.

The purpose of this current evaluation is to compare patient characteristics and smoking cessation outcomes between those who followed through with the preoperative referral and those who did not. Secondarily, we describe the intervention provided (e.g. number and duration of sessions attended, recommended therapy) and determine if session duration and recommended therapy affected smoking cessation outcomes for those who underwent the intervention.

## METHODS

### Setting

Participants were identified from the Edmonton Bone and Joint clinic (EBJ Clinic), which is a centralized preoperative clinic that prepares over 3000 patients annually for their TJR. At the time of our study, 23% of patients attending the clinic were current smokers and the waiting time from surgical decision to the TJR surgery was a median of 25 weeks (unpublished data). This allowed time for a smoking cessation intervention without delaying surgery.

The BJ clinic faxed a referral to a centralized office of a grocery chain that offered pharmacy services in each store. The EBJ clinic left the responsibility for the smoking cessation intervention with the grocery chain’s pharmacies. The pharmacist-led program could occur at any participating pharmacy that was convenient for the participant.

### Study design and population

This prospective evaluation was nested within our previously published comparative study that looked at the fax referral process relative to usual preoperative care for patients who were smokers and booked for TJR^7^. Participants in the ‘pre’ observational phase (OP) received usual care for smoking cessation while ‘post’ intervention phase (IP) participants had a referral sent to a community-based pharmacist-led smoking cessation program. We enrolled 224 (120/150 eligible OP; 104/286 eligible IP) participants. At 6 months post-recruitment, 8 (7%) OP participants self-reported 30-day point prevalence abstinence compared to 21 (20%) IP participants (p=0.003). This current evaluation focused only on those participants referred to the community-based smoking cessation program preoperatively.

All patients scheduled for TJR between April 2015 and August 2016 who were current smokers (any cigarette smoking in the past 30 days), 18 years or older and understood English sufficiently to provide written informed consent were invited to participate. Exclusion criteria were: current major psychiatric disorder, previous suicidal behavior, previous psychotic/mood or psychiatric event associated with smoking cessation and/or active substance use disorder. This study was approved by the human research ethics board at the University of Alberta (Pro00044725); all participants provided signed informed consent.

### Enrollment

We enrolled participants who met the eligibility criteria at the initial consultation with the surgeon that occurred a mean of 12.4±11.6 weeks preoperatively. Participants completed a brief smoking history questionnaire of the past seven and 30 days. Smoking status was validated using exhaled carbon monoxide (CO) monitor (piCO+, Bedfont Scientific Ltd), with a CO breath level of 10 ppm used as a cut-off point to indicate current smoking status^8^.

At enrollment, participants watched a surgeryspecific smoking cessation educational video displayed on a handheld device (https://www.youtube.com/watch?v=Ac84IO4IJkk&feature=you.tube) that included details regarding the availability of a pharmacist-delivered smoking cessation program in their own community for participants. All participants had universal healthcare coverage with free access to the smoking cessation program.

### Pharmacy intervention

Participating pharmacists were trained in smoking cessation in accordance with the provincial standards^9^ (see Supplementary file), and could also independently prescribe smoking cessation medications, including varenicline^10^.

Following enrolment, a fax referral was sent to a centralized office of the grocery chain, which then attempted to contact participants 3 times to set up a smoking cessation consultation with a pharmacist before noting them as non-respondents for the pharmacy consultation. Participants who were contacted by the centralized office were able to select a community pharmacy that was convenient to them. For participants who did not attend the booked pharmacy consultation, the community pharmacist then made 3 additional attempts to rebook the consultation appointment before, again, noting the participant as a non-respondent; thus a maximum of 6 attempts were made to have participants attend the preoperative pharmacy consultation.

For those who met with the pharmacist, a full medication and smoking history review was performed at the initial consultation visit. Pharmacists used an evidence-based smoking cessation intervention booklet developed by the pharmacy chain to provide an individualized smoking reduction and cessation program based on participants’ history and preferences. The overall program included visits with the pharmacist (number of visits was at the participant’s discretion) to review progress and to discuss the information provided in the booklet, which included issues like dealing with nicotine withdrawal, medication side effects and understanding their nicotine dependency. It was not mandatory that participants use smoking cessation medications. Follow-up visits were arranged as the individual participant and pharmacist felt were appropriate with self-reported smoking status recorded at each visit (quit, reduced or no change since program entry). Pharmacists also collected data regarding number of contact attempts, number of visits, time spent with patients, smoking cessation approaches utilized, including medications prescribed when appropriate.

### Outcomes

Smoking cessation was the primary outcome and was ascertained independently of the participating pharmacists. The research associate attempted to contact all participants regardless of whether or not they consulted with a pharmacist; those who were not contacted were assumed to be still smoking. At 6 weeks post-operative, participants completed the brief smoking history questionnaire and smoking status was validated using exhaled CO monitoring. At 6 months post-recruitment, participants completed the smoking history questionnaire via telephone interview.

In addition, pharmacists provided clinic notes so that the number of sessions attended, duration of each session and smoking cessation approach used, including type of medication prescribed when utilized, could be determined. Although we also evaluated self-report of smoking status to the pharmacist (quit, reduced, no change), we only used these data for the descriptive analysis of the smoking cessation program and not as the primary outcome.

### Statistical analysis

Descriptive analyses were performed with participant characteristics at study enrollment compared between those who consulted with the pharmacist and those who did not, using Student’s t-test for continuous variables and chi-squared tests for categorical variables. All patients who were lost to follow-up visits were assumed to be still smoking.

Comparisons were then made between those consulting with a pharmacist and those who did not at: a) 6 weeks post-operatively, and b) again at 6 months post-recruitment using 7-day and 30-day point prevalence abstinence (PPA). At 6 weeks, abstinence was also validated using exhaled CO monitoring.

Secondary descriptive analyses were also performed on those who consulted a pharmacist to examine details of the use of the smoking cessation program. Comparisons were made to determine the impact of recommended therapy (nicotine replacement, varenicline, none) and duration of pharmacist contact. Analyses were performed using the Statistical Program for the Social Sciences (SPSS) version 19.0 (IBM SPSS, Armonk, NY).

## RESULTS

Of 286 smokers invited to participate, only 104 (36%) participated, with 1 (1%) participant subsequently withdrawing. Of 103 participants, 61 (59%) were initially contacted by the centralized office and, of those, 58/103 (56%) subsequently consulted with a pharmacist. For the most part, there were no significant differences between those who saw the pharmacist and those who did not, in term of demographics, health variables, and smoking history (Table 1). However, non-respondents were slightly younger than respondents and had a significantly higher CO status at the baseline assessment (Table 1).

**Table 1:** Baseline characteristics of participants who attended or did not attend a preoperative smoking cessation consultation with a community pharmacist

	*Did not consult with pharmacist preoperatively (N=45)*	*Consulted with pharmacist preoperatively (N=58)*	*p*
**Demographics**			
Gender (female), n (%)	26 (57.8)	28 (48.3)	0.3
Residence (urban), n (%)	34 (75.6)	50 (86.2)	0.2
Age (years), mean (SD)	56.5 (6.7)	60.3 (9.2)	**0.02**
**Health variables**			
Asthma, n (%)	3 (6.7)	5 (8.6)	0.7
Pulmonary disease, n (%)	14 (31.1)	17 (29.3)	0.8
Diabetes, n (%)	12 (26.7)	11 (19.0)	0.4
Heart disease, n (%)	7 (15.6)	8 (13.8)	0.8
Hypertension, n (%)	21 (46.7)	29 (50.0)	0.7
Stroke, n (%)	1 (2.2)	3 (5.2)	0.6
Previous operation, n (%)	43 (95.6)	54 (93.1)	0.6
BMI (kg/m^2^), mean (SD)	32.0 (7.0)	32.2 (6.7)	0.4
**Smoking history**			
Fagerström score, mean (SD)	4.4 (2.2)	3.7 (2.1)	0.1
Cigarette smoked per day, mean (SD)	14.3 (7.5)	13.5 (7.2)	0.6
Years smoked, mean (SD)	36.2 (9.4)	37.5 (12.4)	0.6
Pack-year, mean (SD)	26.5 (16.0)	25.8 (17.2)	0.8
CO level (ppm) at baseline, mean (SD)	23.1 (11.0)	18.7 (7.4)	0.02
At least 1 previous quit attempt, n (%)	42 (93.3)	57 (98.3)	0.2
Ever used smoking cessation medication, n (%)	35 (77.8)	46 (79.3)	0.8

Numbers n (%) are given as percentages of the column population N.

At 6 weeks post-operative, 66/103 (64%) participants returned for re-assessment while at 6 months postrecruitment, 63/103 (61%) participants completed the telephone interview.

The validated 7-day PPA at 6 weeks post-operative in those who consulted the pharmacist was 11/58 (19%) compared to only 2/45 (4%) in those who did not consult the pharmacist (p=0.04). The 30-day validated PPA at 6 weeks was 10 (17%) in the pharmacist-consulting participants and only 1 (2%) in the non-consulting group (p=0.02). At 6 months post-recruitment, 19 (33%) pharmacist-consulting participants self-reported a 7-day PPA compared to only 2 (4%) non-consulting participants (p<0.001) while the 30-day PPA was 19 (33%) in the consulting group relative to 1 (2%) in non-consulting group (p<0.001).

For those initially contacted by the pharmacist (n=58), 52 (90%) returned for a follow-up visit with over half (n=33; 54%) seeing the pharmacist 4 times (Table 2). The mean overall time spent with patients was 71.8±24.4 minutes with no significant difference in time spent with patient between those who quit, reduced or reported no change in their smoking (Table 2). Interestingly, a higher number of participants reported quitting to pharmacists (n=24/58; 41%) than to the research associate (11/58, 19% at 6 weeks; 19/58, 33% at 6 months). In addition to those who reported quitting smoking, an additional 11 (19%) participants of those who saw the pharmacist reported reducing their smoking after the intervention.

**Table 2:** Description of participant use of pharmacistled community-based smoking cessation program

	*n/N (%)*
**Pharmacy centralized office contacted the patient**	61/103 (59.2)
**Number of times patient seen by a pharmacist**	
0	3/61 (4.9)
1	6/61 (9.8)
2	4/61 (6.6)
3	15/61 (24.6)
4	33/61 (54.1)
**Self-reported smoking status at last visit to the pharmacy**	
Quit	24/58 (41.4)
Reduced smoking	11/58 (19.0)
No change in smoking	23/58 (39.7)
	***Mean (SD)***
**Overall mean time spent with patient** (minutes)	71.8 (24.4)
Overall mean time spent with patient by selfreported smoking status at last visit to the pharmacy * (minutes)	
Quit	74.7 (25.0)
Reduced smoking	70.6 (33.7)
No change in smoking	69.5 (18.7)

* F=0.276, p=0.760.

Of those who consulted with a pharmacist, 26 (45%) and 19 (33%) were prescribed nicotine replacement therapy and varenicline, respectively, and 13 (22%) chose not to use any medication. Although no significant difference was seen in the overall selfreported smoking status among these 3 groups at last pharmacist visit, *post hoc* analysis suggested that varenicline was more effective than no medication (p=0.04), but not significantly different from nicotine replacement therapy (p=0.1) (Table 3).

**Table 3:** Self-reported smoking status at last follow-up with pharmacist based on the recommended therapy utilized by participants attending pharmacist-led community-based smoking cessation program

	Varenicline * (N=19)	NRT** (N=26)	*No medication (N=13)*	*p*
**Self-reported smoking status**				0.1
Quit, and still abstinent	11 (57.9)	11 (42.3)	2 (15.4)	
Reduced smoking	3 (15.8)	6 (23.1)	2 (15.4)	
No change in smoking	5 (26.3)	9 (34.6)	9 (69.2)	

Numbers n (%) are given as percentages of the column population N.

* Significant difference compared to ‘No medication’ (p=0.04).

** No significant difference compared to ‘No medication’ (p=0.1).

## DISCUSSION

Our current evaluation, which was nested in a larger comparative study^7^, demonstrated that those participants who complied with the referral to the pharmacist-led, community-based smoking cessation program, had much higher smoking cessation rates than those who did not comply. The individualized intervention, which, on average involved 4 visits with the pharmacist, for just over an hour in total, led to smoking cessation rates of almost 20% at 6 weeks postoperatively that increased to 33% within 6 months of recruitment. Participants who did not consult a pharmacist, despite the referral, reported quit rates of 1–2% in this same time frame. Interestingly, greater than 20% of participants used only behavioral approaches to quit smoking, which appeared to be similarly effective as medication approaches, although those who used varenicline may have achieved higher quit rates than those who chose not to use medication.

Others have suggested that community-based pharmacist-led smoking cessation programs are effective, but no previously published reports of pharmacy programs have specifically been directed at the pre-operative surgical patient population^5,6^. Further, previous studies have shown the benefit of pre- and peri-operative smoking cessation programs, but most have evaluated programs specifically developed for this patient population rather than using pre-existing community-based smoking cessation resources^2,5^. Our intent was not to develop and evaluate a new smoking cessation program, but rather to see if an existing community resource would address the needs of this patient population. Our positive findings support that such programs can be effective; thus, the details provided in this evaluation should prove useful to others considering similar approaches.

Despite the success achieved, we noted that the voluntary participation reduced the numbers of participants who consulted a pharmacist^7^. Since our initial publication, this preoperative process has been deemed mandatory for patients who are current smokers. Patients do not have to cease smoking preoperatively but must attend at least the initial consultation with the pharmacist. Our rationale for this ‘opt out’ approach after the initial consultation is multi-factorial. It ensures that all patients are aware of community-based smoking cessation programs that are covered by our universal healthcare coverage. Quitting smoking is difficult and most patients attempt quitting multiple times before succeeding^11^, so awareness of this resource may facilitate future quit attempts if the patient is not ready to quit at time of surgery. In addition, as noted by others, surgery is a ‘teachable moment’ when patients are aware that smoking may lead to a sub-optimal post-operative outcome, so some patients may consider quitting because they are undergoing a major surgery^12^.

### Strengths and limitations

The current evaluation adds details about the smoking intervention that were not included in our initial publication that compared the impact of embedding a fax referral process relative to usual care. Herein, we discuss the intensity and duration of the pharmacist intervention as well as the approaches used, with *post hoc* analysis suggesting that varenicline may have modest benefits over not using medication. Although pharmacists followed evidence-based smoking cessation guidelines, programs were individualized to address the identified needs and preferences of each participant. Despite allowing program heterogeneity, we still achieved substantial smoking cessation rates relative to those participants who did not consult with pharmacists.

There are some limitations in our study. As previously stated, almost half of participants who agreed to participate in the study did not follow through with the pharmacy consultation despite repeated contact attempts. Further, we utilized only one grocery chain, when we tested this process because they were very willing to develop a standardized approach. Although this chain had provincial coverage, our current program has been extended so that patients can select their own pharmacist (if appropriately trained) or a trained pharmacist within their preferred provider. In addition, self-reported abstinence was used at the 6 months telephone evaluation; however, our data showed that the discrepancy between self-reported and validated smoking status was minimal at 6 weeks post-surgery (data not shown), so it would seem unlikely that there would be significant discordance between self-report and actual status at the 6 months follow-up. Finally, although follow-up over time was lower than anticipated, all participants were retained in the smoking cessation analysis with all those lost to follow-up assumed to be still smoking. This conservative analytic approach ensures that we could not overestimate the impact of the smoking cessation intervention.

## CONCLUSIONS

Our findings show that utilization of an existing community-based pharmacist-led smoking cessation program led to higher smoking cessation rates in patients waiting for TJR surgery, relative to those who chose not to use the program. Thus, use of these types of community resources should be considered part of the standard of care prior to elective surgery.
