# Supplementary material for: A community-based pharmacist-led smoking cessation program, before elective total joint replacement surgery, markedly enhances smoking cessation rates
Source: Tob Induc Dis. 2020 Sep 14;18:78. doi: 10.18332/tid/126405 (PMC7528266; doi:10.18332/tid/126405)
Supplement: Supplementary file 1 [file TID-18-78-s1.pdf]

Q.U.I.T.

QUIT USING & INHALING TOBACCO

RESOURCES FOR THE QUITTER

SELF-HELP RESOURCES (CANADIAN):

- **Canada On-line**
  www.canadaonline.about.com/od/quitsmoking/
  Quit smoking with the help of several Canadian resources, including information on the health benefits of quitting, the health risks of tobacco use and where to find support groups and programs in Canada.
- **Health Canada**
  www.gosmokefree.ca
  Health Canada has a solid collection of information on tobacco use and tobacco control in Canada, health facts and how to quit smoking. It Includes Health Canada's "On the Road to Quitting" self-help program, free quit smoking daily e-mail messages, and "Quit4Life," a program designed to help Canadians ages 12-18 quit smoking.
- **Physicians for A Smoke-Free Canada**
  www.smoke-free.ca
  Provides information and resources on tobacco-related issues to professionals and the public.
- **Canadian Cancer Society**
  www.cancer.ca
  Provides self-help resources for those thinking about and those not thinking about quitting.
- **Heart & Stroke Foundation**
  www.heartandstroke.ca
  Provides information and resources on quitting smoking.
- **Canadian Provincial Lung Associations**
  www.lung.ca
  This site links to all provincial lung associations and provides information and resources on how to quit and where to get support.

To access resources specific to your province or territory, contact your local lung association, cancer society, heart and stroke foundation, drug dependency services or local smokers' helpline.

SELF-HELP RESOURCES (INTERNATIONAL):

- **Quit Net**
  www.quitnet.org
  This is one of the best resources for on-line quit smoking support. Peer to peer support, quitting strategies, Q-gadget-tracks lifetime and money saved, expert counsellors, personal profiles, anniversary e-mails and lots more!
- **Nicotine Anonymous**
  www.nicotine-anonymous.org
  A non-profit 12 Step Fellowship of men and women helping each other live nicotine-free.
- **Why Quit?**
  www.whyquit.com
  This website is a little heavy-handed and goes into the grisly zone but features a large menu of resources related to tobacco-cessation.
- **On-line Chat Room**
  www.quitsmokingsupport.com
  On-line chat rooms and bulletin boards for people who want to quit smoking.

© 2006 Canadian Pharmacists Association

Canadian Pharmacists Association
Association des Pharmaciens du Canada

Q.U.I.T.

QUIT USING & INHALING TOBACCO

QUIT TIPS & TRICKS

MANAGING CRAVINGS/TRIGGERS

- Chew on a toothpick
- Chew on a clove
- Munch on raw veggies
- Drink ice water
- Play cards
- Doodle
- Watch television
- Get a breath of fresh air
- Brush and floss your teeth
- Splash cold water on your face
- Take a warm shower
- Hull sunflower seeds
- Chew on a straw
- Peel a large carrot and eat it slowly
- Sleep in
- Chew gum
- Go for a brisk walk
- Knit/cross-stitch
- Work on a crossword puzzle
- Shell peanuts
- Fold laundry
- Chew on licorice root

STRESS RELIEF

- Deep breathe
- Organize days to avoid tension especially around quit day
- Be physically active
- Take relaxation breaks

ENVIRONMENTAL CONTROL

- Make smoke-free zones (e g , home, car, garage)
- Get rid of all tobacco products and accessories such as matches, lighters and ashtrays
- Clean and deodorize your home
- Rearrange a room in your home
- Stock your fridge with healthy snacks
- Clean and deodorize your car
- Take a different route to work
- Put gum, sugar-free candies or mints in your car ashtray
- Put a no-smoking sign in your car
- Put a picture of a loved one where you once kept your cigarettes

SOCIAL SUPPORT

- Choose your quit supporters with care. Choose people you know have your best interest at heart
- Remind friends and family that you are quitting and ask for their support
- Talk to friends who have quit smoking
- Let friends and family know that you will come to them if you require their support
- Give friends and family specific examples of how they can support you
- Phone a friend
- Enlist a quitting buddy

4 D's

- Delay
- Do Something Different
- Drink Water
- Deep Breathe

THOUGHT MANAGEMENT / MOTIVATION

- Review your reasons for quitting.
- Tell yourself:
  - "I can do this. I am worth it."
  - "I can do this. People quit every day."
  - "I've overcome other big obstacles in my life."
  - "Smoking is not an option right now."
  - "I'm a puff away from a pack a day."
  - "I'm gaining my health and control over my life."
- Reward yourself, rent a movie, buy something nice
- Calculate the amount of money you are saving
- Visualize yourself as a non-smoker

SOCIALIZING

- Avoid friends who smoke for a while
- Prepare and rehearse a standard line for refusing cigarettes
- Have an escape plan. Leave a risky situation by going for some fresh air, to the washroom, etc., until you regain control

© 2006 Canadian Pharmacists Association

CANADIAN PHARMACISTS ASSOCIATION
ASSOCIATION DES PHARMACIENS DU CANADA

Q.U.I.T.
QUIT USING & INHALING TOBACCO

SMOKING REDUCTION TIPS

- ✔ Keep a smoking record of when and why you smoke. Then, gradually cut out the cigarettes you smoke, from least to most important.
- ✔ Set a daily quota of cigarettes. Put only this number in your pack in the morning.
- ✔ Delay your first cigarette of the day by half an hour.
- ✔ Delay smoking for 15 minutes whenever you have a craving. Taking deep breaths or chewing gum also helps.
- ✔ Smoke only half of each cigarette.
- ✔ Keep your pack in an inconvenient place, like the cupboard above the fridge or in the closet.
- ✔ Wrap your cigarette pack with a rubber band or string. Unwrapping it every time you smoke will remind you that you're trying to quit.
- ✔ Stop whatever you're doing — even driving — when you have a cigarette, and think only about your smoking.
- ✔ Have a practice quit day. Stop smoking for 24 hours.
- ✔ Avoid situations in which you usually smoke and plan activities that don't involve smoking. For example, spend time with your kids without a cigarette.
- ✔ Brush your teeth often, especially during a craving.
- ✔ Keep on hand celery or carrot sticks, sugarless gum, ...
- ✔ Drink lots of water (6-8 glasses per day).
- ✔ If you roll your own cigarettes, roll only a few at a time.
- ✔ Change the brand you smoke each time you buy a pack.
- ✔ Smoke with the opposite hand.

Adapted from *Stop Smoking: A Program for Women*, Canadian Public Health Association, 1999

© 2006 Canadian Pharmacists Association

Canadian Pharmacists Association | Association des Pharmaciens du Canada

Q.U.I.T.

QUIT USING & INHALING TOBACCO

FREE TELEPHONE ADVICE

QUIT LINES

Quit lines offer free telephone support from trained specialists who can help develop a personal quit plan or provide counselling to family and friends of smokers. They can also answer any questions you may have and locate self-help materials and other quit smoking support in your community.

- **Newfoundland and Labrador Smokers' Helpline**
  1-800-363-5864 M-T 9:00 am-9:00 pm, F 9:00 am-5:00 pm, 24/7 voicemail
  Service provided in English
  www.smokershelp.net; info@smokershelp.net
  Speak with a counsellor within hours of service
  Mailed information or self-help resources
  Proactive quit smoking counselling
  Reactive quit smoking counselling
  Referral to other services

- **New Brunswick Smokers' Helpline**
  1-877-513-5333
  M-T 8:00 am-8:00 pm, F 8:00 am-4:00 pm, 24/7 voicemail
  Service provided in English and French
  www.cancer.ca; ccsnb@nb.cancer.ca
  Speak with a counsellor within hours of service
  Mailed information or self-help resources
  Proactive quit smoking counselling with scheduled follow-up
  Reactive quit smoking counselling
  Referral to other services

- **Nova Scotia Smokers' Helpline**
  1-877-513-5333
  M-T 8:00 am-8:00 pm, F 8:00 am-4:00 pm, 24/7 voicemail
  Service provided in English and French
  www.cancer.ca; ccs.ns@ns.cancer.ca
  Speak with a counsellor within hours of service
  Mailed information or self-help resources
  Proactive quit smoking counselling with scheduled follow-up
  Reactive quit smoking counselling
  Referral to other services

- **Prince Edward Island Smokers' Helpline**
  1-888-818-6300
  M-T 8:00 am-8:00 pm, F 8:00 am-4:00 pm, 24/7 voicemail
  Service provided in English and French
  www.cancer.ca; info@pei.cancer.ca
  Speak with a counsellor within hours of service
  Group cessation programs
  Mailed information or self-help resources
  Proactive quit smoking counselling with scheduled follow-up
  Reactive quit smoking counselling
  Referral to other services

- **Québec: Ligne j'Arrête**
  1-866-jarrête (527-7388)
  M-F 7:30 am-8:00 pm, 24/7 voicemail
  Service provided in French and English
  www.jarrete.qc.ca; webmestre@quebec.cancer.ca
  Speak with a counsellor within hours of service
  Mailed information or self-help resources
  Proactive quit smoking counselling with scheduled follow-up
  Reactive quit smoking counselling
  Referral to other services
  Email information and support
  Online chat
  Quit to win challenge

- **Ontario Smokers' Helpline**
  1-877-513-5333
  M-T 9:00 am-9:00 pm, F 9:00 am-5:00 pm, 24/7 voicemail
  Service provided in English and French
  www.cancer.ca; ontdiv@ontario.cancer.ca
  Speak with a counsellor within hours of service
  Mailed information or self-help resources
  Proactive quit smoking counselling with scheduled follow-up
  Reactive quit smoking counselling
  Referral to other services

- **Manitoba Smokers' Helpline**
  1-877-513-5333
  M-T 10:00 am-10:00 pm, F 10:00 am-6:00 pm, 24/7 voicemail
  Service provided in English and French
  www.cancer.ca; info@mb.cancer.ca
  Speak with a counsellor within hours of service
  Mailed information or self-help resources
  Proactive quit smoking counselling with scheduled follow-up
  Reactive quit smoking counselling
  Referral to other services

- **Saskatchewan Smokers' Helpline**
  1-877-513-5333
  M-T 10:00 am-10:00 pm, F 10:00 am-6:00 pm, 24/7 voicemail
  Service provided in English and French
  www.cancer.ca; ccssk@sk.cancer.ca
  Speak with a counsellor within hours of service
  Mailed information or self-help resources
  Proactive quit smoking counselling with scheduled follow-up
  Reactive quit smoking counselling
  Referral to other services

- **Alberta Smokers' Helpline**
  1-866-332-2322
  8:00 am-8:00 pm, 7 days a week
  Service provided in English
  www.aadac.com; tru@aadac.gov.ab.ca
  Speak with a counsellor within hours of service
  Web based information
  Mailed information or self-help resources
  Proactive quit smoking counselling with scheduled follow-up
  Reactive quit smoking counselling
  Referral to other services

- **British Columbia/Yukon: Quit Now By Phone**
  1-877-455-2233
  24/7 for incoming calls
  Service provided in English, French and 121 other languages
  www.quitnow.ca; info@bc.lung.ca
  Speak with a counsellor within hours of service
  Mailed information or self-help resources
  Proactive quit smoking counselling with scheduled follow-up
  Reactive quit smoking counselling
  Referral to other services

- **Northwest Territories: Quit Now By Phone**
  1-866-353-03383
  www.dontbeabutthead.ca; dontbeabutthead@taitcc.com

- **Nunavut: Quit Now By Phone**
  1-866-877-3845

© 2006 Canadian Pharmacists Association

CANADIAN PHARMACISTS ASSOCIATION — ASSOCIATION DES PHARMACIENS DU CANADA
